# Weathering the storm: parental effort and experimental manipulation of stress hormones predict brood survival

**DOI:** 10.1186/s12862-015-0497-8

**Published:** 2015-10-05

**Authors:** JQ Ouyang, ÁZ Lendvai, R. Dakin, AD Domalik, VJ Fasanello, BG Vassallo, MF Haussmann, IT Moore, F. Bonier

**Affiliations:** Department of Biological Sciences, Virginia Tech, Blacksburg, USA; Department of Animal Ecology, Netherlands Institute of Ecology (NIOO-KNAW), Wageningen, The Netherlands; Department of Evolutionary Zoology and Human Biology, University of Debrecen, Debrecen, Hungary; Department of Zoology, University of British Columbia, Victoria, BC Canada; Department of Biology, Queen’s University, Kingston, Canada; Department of Biology, Bucknell University, Lewisburg, USA; Present address: NIOO-KNAW, P.O.Box 50, , 6700 AB Wageningen, The Netherlands

**Keywords:** Corticosterone, Stress, Reproduction, Tree swallow, Tachycineta bicolor, Biparental care, Inclement weather, Hormone implant

## Abstract

**Background:**

Unpredictable and inclement weather is increasing in strength and frequency, challenging organisms to respond adaptively. One way in which animals respond to environmental challenges is through the secretion of glucocorticoid stress hormones. These hormones mobilize energy stores and suppress non-essential physiological and behavioral processes until the challenge passes. To investigate the effects of glucocorticoids on reproductive decisions, we experimentally increased corticosterone levels (the primary glucocorticoid in birds) in free-living female tree swallows, *Tachycineta bicolor*, during the chick-rearing stage. Due to an unprecedented cold and wet breeding season, 90 % of the nests in our study population failed, which created a unique opportunity to test how challenging environmental conditions interact with the physiological mechanisms underlying life-history trade-offs.

**Results:**

We found that exogenous corticosterone influenced the regulation of parental decisions in a context-dependent manner. Control and corticosterone-treated females had similar brood failure rates under unfavorable conditions (cold and rainy weather), but corticosterone treatment hastened brood mortality under more favorable conditions. Higher female nest provisioning rates prior to implantation were associated with increased probability of brood survival for treatment and control groups. However, higher pre-treatment male provisioning rates were associated with increased survival probability in the control group, but not the corticosterone-treated group.

**Conclusions:**

These findings reveal complex interactions between weather, female physiological state, and partner parental investment. Our results also demonstrate a causal relationship between corticosterone concentrations and individual reproductive behaviors, and point to a mechanism for why naturally disturbed populations, which experience multiple stressors, could be more susceptible and unable to respond adaptively to changing environmental conditions.

## Background

How much time and energy should parents invest in their offspring? This question has long fascinated evolutionary ecologists because the answer links major fields of life-history theory, sexual selection, and population ecology [1–3]. The concept of parental investment is based on the observation that reproduction is costly [4]; therefore, allocation of resources towards current reproduction can come at a cost to longevity and future reproduction [2]. However, the optimal balance of this trade-off strongly depends on environmental context: complex or unpredictable environments can alter the fitness costs associated with parental investment and thereby influence the evolution of life-history strategies. Current environmental conditions are changing rapidly, and population persistence may be influenced by the capacity of organisms to respond flexibly to these changes [5].

Investigating endogenous hormone levels can provide exceptional insight into the proximate mechanisms mediating context-dependent resource allocation decisions because hormone levels change in response to both environmental and internal stimuli [6–10]. Glucocorticoid steroid hormones (GCs) are particularly important because they maintain energetic balance in the face of changing environmental demands [11–14]. The function of GCs varies with circulating concentrations [15, 16]. For instance, at elevated levels (Level C sensu [17, 18]), GCs induce an emergency life-history stage and promote physiological and behavioral responses that enhance immediate survival while inhibiting reproduction [19]. In contrast with the negative effects of high GC levels on reproduction, at seasonal baseline levels (Level B sensu [17, 18]), GC levels are positively related to reproductive performance, and help sustain the high metabolic costs of parental care [20, 21]. For example, recent studies that experimentally elevated corticosterone (‘cort’, the primary GC in birds) levels within the baseline range found support for a positive influence of baseline cort on reproductive effort, such as increases in incubation and parental care [22–24]. Thus, the ‘cort-adaptation hypothesis’ suggests that individuals that can effectively up-regulate their endogenous cort secretion will meet the high energetic requirement of parental care [25]. The opposing effects that cort exerts on survival and reproduction make GCs ideal candidates as a common physiological mechanism underlying life-history variation [14, 26, 27], but we lack experimental studies in free-living species under varying environments.

Our goal in this study was to investigate the effects of elevated cort levels on parental investment in breeding birds. We used exogenous cort pellets to experimentally increase cort levels in female tree swallows (*Tachycineta bicolor*) during chick-rearing. The study year (2013) was characterized by a period of exceptionally cold and rainy weather at our field site, which had a detrimental impact on the flying insects that tree swallows rely upon exclusively to feed their offspring [28]. Repeated, sudden drops in temperature led to massive reproductive failure in the study population: 90 % of tree swallow nests failed, as opposed to an average of 53 % nest failure in this population over the previous 5 years [F. Bonier unpublished]. Although the immediate cause of this mortality remains unknown (adults may have deserted the brood, or the young could have died due to insufficient food supplies and/or hypothermia), harsh weather conditions throughout the season could have exerted strong selection on the combination of traits that enabled some individuals to cope with weather as nest desertion occurred throughout the season. Thus, our study represents a unique investigation of the combined effects of parental feeding rates prior to hormone manipulation and experimentally elevated cort levels on reproductive success under varying environmental conditions within a breeding season. We investigated how adverse weather conditions interacted with the experimental cort elevation to affect brood mortality. In addition, we investigated whether initial parental feeding rates were associated with brood survival and whether the hormonal treatment and weather conditions altered this effect.

## Results

There were no significant differences (all p-values > 0.2) between the control and treatment groups in pre-manipulation cort levels, body condition, hematocrit, brood size, or feeding rates. Thirty-one of 33 nests (15 of 16 cort-female nests, 16 of 17 control-female nests) failed during the nestling period, although timing of nest failure varied. Because we captured adults with nest box traps, and because swallows depart from the study site after nest failure, we were not able to measure post-treatment hormone levels.

Parental body condition and pre-implant cort levels (male and female) did not predict brood survival time (all *p* > 0.16). Average nestling mass also did not predict brood survival time: on average, a one gram increase in nestling mass decreased daily mortality by 6 %, but this effect was not significant (*p* = 0.69). Brood size also did not predict brood survival (*p* = 0.36).

**Fig. 1:**
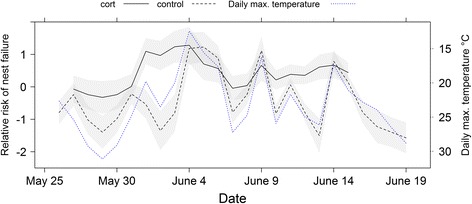
Rapid, repeated drops in temperature (upward spikes in the dotted line) were accompanied by concomitant increases in mortality, especially in control broods. Predicted values (relative risk of nest failure, i.e., daily mortality relative to the mean) and corresponding standard errors from the best-supported Cox proportional hazard model (see Table 2a) are plotted against date. The grey bands represent the standard error of the predicted values for the risk of nest failure (*left y-axis*). The dotted blue line shows the daily maximum temperature (°C) and corresponds to the right *y-axis*. To facilitate interpretation, the scale on the right y-axis was reversed

**Fig. 2:**
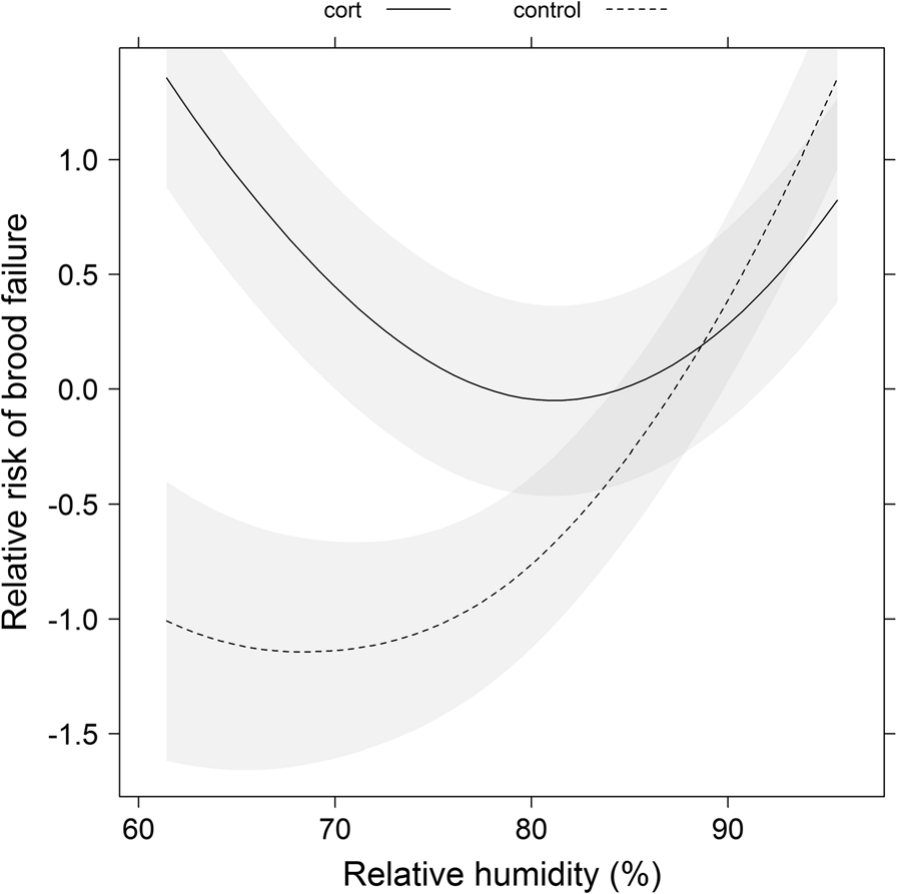
When relative humidity is low, broods of cort-implanted females had higher risk of failure than control broods, but this difference disappears when relative humidity is high. Predicted values (relative risk of nest failure, i.e., daily mortality relative to the mean) from the best-supported Cox proportional hazard model (see Table 2a) are plotted in relation to relative humidity and the treatment. Solid and dashed lines represent cort and control groups, respectively, and the grey bands represent the standard error of the predicted values. A loess smoother was applied to the predicted values to facilitate interpretation. The non-linearity of the predictions is due to the effect of maximum temperature in the model

We examined how weather altered the effect of treatment on brood survival and found that maximum temperature and relative humidity significantly improved model fit (Table 1a). The best-supported model in this set of candidate models included maximum temperature and an interaction between relative humidity and treatment (Table 1a). Parameter estimates (Table 2a) showed that broods of cort-implanted females had higher failure risk (i.e., higher daily mortality) than control broods. However, this effect was modified by weather. Cold temperatures increased daily mortality, such that decreases in daily temperature induced immediate, concomitant increases in nestling mortality (Fig. 1). A strong interaction effect between treatment and relative humidity was observed: when relative humidity was low (dry weather, daily humidity below 80 %), cort treatment increased the risk of brood failure compared to controls, but this difference disappeared during humid weather (relative humidity above 80 %; Fig. 2).

**Table 1:** Candidate model sets for A) the effect of weather and cort treatment, B) the effect of parental behavior and cort treatment, and C) the effect of weather, parental behavior, and cort treatment on tree swallow brood survival

	Model id	K	AICc	Effects in the model	ΔAICc
A.	1	4	160.93	maxtemp + rh × treatment	0.00
	2	5	162.82	(maxtemp + rh) × treatment	1.89
	3	2	163.34	maxtemp + treatment	2.41
	4	3	165.34	maxtemp + rh + treatment	4.41
	5	2	165.86	maxtemp + rh	4.93
	6	4	166.40	rh + maxtemp × treatment	5.47
	7	2	167.93	rh + treatment	7.00
	8	1	168.33	treatment	7.40
	9	0	168.72	null	7.79
B.	10	4	163.37	ffv + mfv × treatment	0.00
	11	5	164.65	(ffv + mfv) × treatment	1.28
	12	4	167.30	mfv + ffv × treatment	3.93
	13	3	167.83	mfv + ffv + treatment	4.46
	14	2	169.63	ffv + treatment	6.27
	15	2	170.25	mfv + treatment	6.88
C.	16	8	159.92	maxtemp × mfv × treatment + ffv	0.00
	17	8	161.92	rh × mfv × treatment + ffv	2.00

*K* denotes the number of parameters in the model, *AICc* refers to the second order Akaike Information Criterion, *ΔAICc* shows the difference between the *AICc* value of a given model and the best-fit model in the set. Variable abbreviations: *maxtemp* daily maximum temperature, *rh* relative humidity, *mfv/ffv* male/female feeding rate (visits/h/chick)

**Table 2:** Parameter estimates from time-dependent Cox proportional hazard models analyzing factors affecting daily chick mortality for the best-fit candidate models from Table 1. Treatment effects are given for control-implanted females relative to cort-implanted females

Variable	coef	SE	*p* value
A. Log rank test score = 14.33, df = 4, *p* = 0.006			
Maximum daily temperature	−0.11	0.05	0.050
Relative humidity	−0.03	0.02	0.187
Treatment (control)	−7.42	2.92	0.011
Relative humidity × treatment	0.08	0.03	0.017
B. Log rank test score = 13.51, df = 4, *p* = 0.009			
Female feeding rate per chick	−0.80	0.32	0.014
Male feeding rate per chick	1.00	0.39	0.010
Treatment (control)	1.11	0.72	0.126
Male feeding rate × treatment	−1.53	0.62	0.013
C. Log rank test score = 25.38, df = 8, *p* = 0.001			
Daily max. temperature	0.17	0.12	0.163
Male feeding rate	5.62	2.24	0.012
Treatment (control)	9.14	3.55	0.010
Female feeding rate	−0.64	0.33	0.053
Daily max. temperature × male feeding rate	−0.22	0.10	0.035
Daily max. temperature × treatment	−0.40	0.18	0.023
Male feeding rate × treatment	−7.59	2.96	0.010
Daily max. temperature × male feeding rate × treatment	0.03	0.15	0.043

*Coef*’ indicates the estimated coefficient and ‘*SE* standard error of that estimate in the model

Second, we looked at whether parental behavior the day before capture predicted brood survival. The best-fit model in this set of candidate models included both male and female feeding rate and an interaction between male feeding rate and treatment (Table 1b). High female feeding rate prior to implantation was associated with lower brood mortality, regardless of implant type (Table 2b). The association between male feeding rate and brood survival was more complex. Nests where males had a high feeding rate prior to implantation had lower brood mortality if the female was subsequently control-implanted, but higher brood mortality if the female was cort-implanted (Table 2b).

**Fig. 3:**
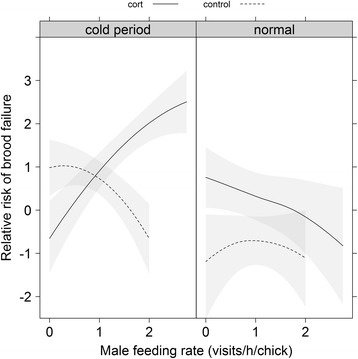
High male feeding rate prior to cort treatment combined with cold temperatures increases the risk of brood failure. Predicted values (relative risk of nest failure, i.e., daily mortality relative to the mean) from the best-supported Cox proportional hazard model (see Table 2c) with respect to pre-treatment male feeding rate and female cort manipulation. Solid and dashed lines represent cort and control groups, respectively, and the grey bands represent the standard error of the predicted values. For visualization purposes in this figure only, maximum daily temperatures <20 °C were considered a cold period and maximum daily temperatures ≥ 20 °C were considered normal; note that temperature was treated as a continuous variable in all statistical analyses (e.g., Table 2c)

Finally, we investigated whether the treatment-dependent effects of male parental behavior on brood survival could be due to a difference between treatment groups in the males’ sensitivity to changing weather conditions (i.e., weather × feeding rate × treatment interaction). For both weather variables (maximum daily temperature and relative humidity), adding this three-way interaction increased model fit substantially (Table 1c). Parameter estimates for these models indicate that during inclement weather (low temperature), initially high male feeding rate was associated with low nestling mortality in control broods, whereas high male feeding rate was associated with high nestling mortality for broods of cort-implanted females. In more favorable weather conditions, initially high male feeding rate was associated with decreased nestling mortality in the cort-implanted group, whereas male feeding rate did not have a consistent relationship with nestling mortality in the control group (Table 2c, Fig. 3).

## Discussion

Inclement weather provided us with an opportunity to test the consequences of an altered physiological state during a major ecological challenge. Our results reveal that the effect of cort treatment on brood survival depends strongly on weather conditions, such that treatment differences were only evident during periods of benign weather. Moreover, our results reveal a complex interaction between weather, the females’ physiological state, and their partners’ parental feeding rate. In particular, male sensitivity to changes in both the environmental conditions and the state of their partner seems to be an important component of the reproductive success of a breeding pair. These findings not only indicate that the causal relationship between cort concentrations and brood survival is context-dependent, but also provide evidence that glucocorticoid mechanisms can attune individual state to external conditions.

It is important to note that we would not have seen the context-dependent effect of cort on reproductive success had we performed the study in a year with typical weather conditions. Repeated drops in temperature (Fig. 1) gave us the ability to analyze how weather affected the daily probability of brood survival. Our results show that low temperatures and high humidity hastened brood mortality for both control and cort-implanted birds, but as weather conditions worsened (e.g., between June 4 and June 7), the difference between treatment groups diminished. This interaction effect was especially marked for relative humidity, resulting in a statistically significant interaction with treatment (Fig. 2). When daily humidity was below 80 %, the risk of brood failure was higher for cort-treatment birds than for controls (Fig. 2). However, this treatment effect disappeared with high relative humidity (> than 80 %; [29]), such that in rainy weather, all individuals had a high risk of brood failure (Fig. 2; Table 2a). These inclement conditions occur in approximately 5 % of breeding seasons in a nearby population [28], and have thus likely shaped selection on reproductive trait reaction norms. Given that extreme weather conditions are predicted to occur more often as a result of global climate change (IPCC AR4), our findings have important implications for understanding whether organisms can respond to increasingly frequent climatic challenges. Individuals with high cort levels may not be able to properly attune their state and reproductive decisions to increasingly challenging environmental conditions.

A limitation of our study is that we were not able to obtain post-implant hormone levels, because catching the adults requires an active nest, and all but three broods had failed by the time of the planned recapture of the parents. Although knowing exactly how the pellets modified the cort profile of the birds would have been useful, we found a difference between the treatment and the control groups in brood mortality, which suggests a causal role of the hormone treatment. Although our conclusions are limited by the lack of post-treatment hormone samples, we can still conclude that the corticosterone implant resulted in a mismatch between environmental conditions and physiology that ultimately caused lower fitness under otherwise favorable conditions.

We found that broods were less able to persist when the females’ cort levels were experimentally increased. The females’ hormonal state is, therefore, an important factor in determining a brood’s susceptibility to environmental fluctuations. In other words, cort-treated females’ brood success resembles that of control females during poor weather conditions. Therefore, elevation of cort appears to increase the parents’ sensitivity to further challenges [30], and/or inhibit individual attunement to weather conditions. Moreover, elevated corticosterone concentrations have been shown to decrease prolactin levels and parental care [31]. Even if moderately elevated cort levels are beneficial for fueling demanding parental activities (as the ‘cort-adaptation hypothesis’ suggests [25]), such elevation may represent a risk-prone strategy, in which the benefits depend on predictable (e.g., climatic) environmental conditions. If this interpretation is correct, then we would expect selection to act against this strategy in environments where the probability of adverse, unpredictable conditions is high. With global climate change, the occurrence of such conditions is increasing ([32]; IPCC AR4), which could in turn affect the evolution of hormonal regulation of resource allocation.

We also found that brood survival was related to initial male provisioning behavior and its interaction with hormone treatment, even though only adult females received treatment implants. While high female parental effort before treatment increased brood survival across both treatment groups, male feeding effort had a positive effect at control nests only. The pattern was reversed for nests where the female was implanted with cort; at these nests, high male investment prior to treatment was associated with lower brood survival. Why would initially high male investment result in lower brood survival in this context? We propose three alternative, but not mutually exclusive, explanations. First, high-invested males may be especially sensitive to changes in their partner’s effort [33, 34]. Male and female feeding rates were positively correlated before treatment [35], so it is possible that males match their feeding effort to their partner’s effort [36]. Therefore, the observed pattern of nest failure might have occurred if cort-treated females decreased their feeding rate, and males with high parental effort responded more strongly to changes in their partner’s effort than other males. Second, males that were investing the most effort early on might be more sensitive to weather changes [37, 38], and less able to persist with their high level of investment after their partner was hormone-implanted. To investigate this possibility, we examined the interaction between male feeding, treatment, and weather conditions. Consistent with this explanation, we found that the opposing effects of initial male (but not female) feeding rates on brood survival were only apparent during unfavorable weather conditions (Fig. 3). Finally, females may be more willing to decrease feeding rate when paired with a good provider. If so, cort-implanted females might be more likely to desert their broods when paired with a high-provisioning male, because a high-provisioning male could potentially provide sole care. Additional studies on parental care dynamics are needed to differentiate among these explanations, to help understand how changing environmental conditions influence the negotiation over parental care.

Our results have implications for understanding and predicting the persistence of breeding bird populations. In general, parents are predicted to stop investing in their current offspring when the marginal gain from parental care is less than that of alternative behaviors that enhance the parent’s future reproductive opportunities [3, 39, 40]. One of the main alternatives to parental care is re-mating and subsequent reproduction, however, the tree swallows in our population do not re-nest later in the same season. Therefore, brood desertion entails zero reproductive success for a given year, potentially reducing lifetime reproductive success (average lifespan of tree swallows 2.7 years; [41]), and contributing to local population decline [42]. With lower temperatures and heavier rainfall in June as compared to previous years, the assessment of physiological mediators of reproductive success and their flexibility in response to external conditions becomes vital.

Our study provides evidence for a causal link between cort and a metric of reproductive success that represents a major component of fitness. Furthermore, our results demonstrate how an individual’s physiological state (cort treatment) can interact with partner parental investment to influence brood survival in response to challenging environmental conditions. We propose that elevated cort levels caused a mismatch between parents’ reproductive behavior and the weather conditions. These results suggest that populations in which organisms experience increased cort levels as a result of disturbance (e.g., urban noise, pollution, and predation) might be less able to respond appropriately to climate change.

## Methods

### Study area and general field procedures

We conducted this study from May 1 to July 30, 2013 at the Queen’s University Biological Station in Elgin, Ontario, Canada (44.6°N, 76.3°W, ~140 m elevation). At this site, there are 229 nest boxes set up in a grid network across nine fields. We monitored tree swallow nests daily following the completion of nest building to determine first egg date (May 8–16th), clutch size, and the onset of incubation. We also checked nests daily 13 days after clutch completion to determine the date of hatching (day 0 of the nestling period), and we weighed the nestlings on day 4 post-hatching. Nests were monitored every other day during chick-rearing. We defined outcome date as the date when the young fledged or were found dead (June 1–June 29). The date when broods were found to have no remaining living chicks (defined as the date of nest failure) was used for brood survival analysis.

Our study period was characterized by unusually high precipitation and rapid, repeated drops in temperature (when daily maximum temperatures fell below 20 °C). According to Environment Canada (https://weather.gc.ca/canada_e.html), total precipitation in June 2013 was 132 mm as compared to only three times in the past 20 years in which rainfall during June exceeded 100 mm. Additionally, for 10 of the 30 days in June, the maximum daily temperature did not reach 18.5 °C, the critical temperature for insect flight [28], as compared to the previous 20 years with an average of only 2.8 days during June with maximum temperatures below 18.5 °C.

All procedures followed guidelines for animal care outlined by ASAB/ABS and the Canadian Council on Animal Care (CCAC), and were approved by the Virginia Tech’s Institutional Animal Care and Use Committee (protocol #12-020) and Queen’s University Animal Care Committee (2013–019). All birds were banded under Canadian Wildlife Services banding permit #10771.

### Behavioral observations

Observers, blind to the experimental treatment, performed behavioral observations for one hour between 0730 and 1300 h during day 3 post-hatching and recorded parental feeding rates (visits/hr/chick) and time spent in the box as a proxy for brooding (durations > three minutes were defined as brooding). In tree swallows, the number of visits to a nest is an accurate measure of food delivered to nestlings [43]. Rose [44] found that the cumulative number of tree swallow parental feeding visits increased linearly over the day, indicating that diel variation in visit behavior is unlikely to affect our estimates of feeding rates. One hour observation of visit rate is a good predictor of total daily visit rate for both females and males in this population [Lendvai et al., MS in review].

### Blood sampling and hormone manipulation

On day 4 of chick-rearing (May 31–June 8), we captured both parents in their nest box and collected a blood sample from the brachial vein (200 μl) within 3 min of capture (mean ± standard error: 2.13 ± 0.003 min). Sample time within this 3-min period was not significantly related to measured cort level (Pearson’s correlation: *r* = 0.12, *p* = 0.13, *n* = 65). Blood was stored in heparinized capillary tubes on ice and sealed with clay until further processing. Samples were centrifuged within 3 h of collection for 10 mins. Hematocrit (relative amount of red blood cells in the total blood volume) was measured to the nearest mm. Red blood cells and plasma were then separated and stored at −20 °C.

After collecting the blood sample, we measured the birds’ skull length (head + bill) to the nearest 0.1 mm, and body mass to the nearest 0.25 g. We released adult males after taking these measurements. Adult females were randomly assigned to either a control or treatment group and received either a cort (*n* = 16) or a control (*n* = 17) pellet implant (0.5 mg 60-day release, Innovative Research of America, Sarasota, FL, USA) of the same type used in previous hormone manipulation experiments with birds [45]. After applying topical lidocaine (10 %) to the exposed skin, we placed subcutaneous implants after making a 2 mm incision on the dorsal epidermis between the shoulders. After the pellet was inserted, we sealed the opening with liquid bandage. Adults were released within 10 min of capture. We allocated treatment using a randomized block design, with capture day as a blocking factor to control for potential seasonal effects.

### Hormone analysis

Total cort from plasma samples was quantified through direct radioimmunoassay, as described by [20]. Briefly, cort was extracted from plasma using dichloromethane, and extracts were reconstituted in PBS buffer. Mean extraction efficiency was 73 % and final concentrations were corrected for individual extraction efficiencies. We used a commercial antiserum (Esoterix Endocrinology, Calabasas Hills, CA 91301, Product number: B3-163). The extracts were incubated overnight at 4 °C with 14 K dpm of 3H-Cort (Perkin Elmer, Product number: NET399250UC) and antiserum. Dextran-coated charcoal was added to separate cort bound to antibodies. After centrifugation, the radioactivity of the bound fraction was counted in a liquid scintillation counter. Within-assay variation among replicate known-concentration standard samples was 3.12 %. Minimal detectable cort levels were 1.16 ng/mL, and no samples fell below this detection limit.

### Statistical analysis

We performed statistical analyses using R version 3.0.1 (R Core Team 2013). We compared pre-implant cort levels, adult body condition, haematocrit, and brood size between the treatment groups. Body condition was estimated as a scaled mass index [46], using each individual’s body mass and skull length. Brood survival was analyzed using Cox proportional hazards regression models for censored data [47–49] with weather conditions as time-dependent covariates (see below for weather measurements). This type of survival analysis models the time that passes before some event occurs in relation to one or more covariates. In our study, the response variable in this analysis was the time until nest failure. We checked nests for brood survival until 20 days post-hatch, and analyzed daily brood survival from implant date until day 20 of the nestling period or nest failure, whichever came first, for all nests in the population (not just for the one nest in each treatment group that fledged offspring). To avoid confounding brood survival with fledging time, the two broods that were still alive at day 20 were right-censored in all analyses of brood survival (broods can fledge at 21 days, range 21–25; F. Bonier unpublished).

We considered the following independent variables and their interactions: hormone treatment, weather conditions, and parental feeding rates (visits/hr/chick), and we used the second order Akaike’s Information Criterion (AICc) to compare candidate models [50]. All models met the assumptions for Cox regression.

Weather data were collected from two HOBO™ data loggers placed at Queen’s University Biological Station that recorded local temperature and relative humidity values every hour. We calculated daily maximum temperature and daily mean relative humidity (as a proxy for precipitation) because previous work on tree swallows indicates that these factors affect parental feeding rate [28, 43]. Both variables were analyzed as continuous variables in our statistical models. Because we checked nests in the morning, nestling survival was likely to be more strongly affected by the previous day’s conditions rather than the conditions on the outcome day. Thus, when analyzing brood survival, we used weather conditions from the preceding day.

## References

[1] Trivers RL, Campbell B (1972). Parental investment and sexual selection. Sexual selection and the descent of man, 1871–1971.

[2] Stearns SC (1992). The evolution of life histories.

[3] Kokko H, Jennions MD (2008). Parental investment, sexual selection and sex ratios. J Evol Biol.

[4] Williams GC (1966). Natural selection costs of reproduction and a refinement of Lack's principle. Am Nat.

[5] Both C, Visser ME (2001). Adjustment to climate change is constrained by arrival date in a long-distance migrant bird. Nature.

[6] Sinervo B, Licht P (1991). Hormonal and physiological control of clutch size, egg size, and egg shape in side-blotched lizards (*Uta-stansburiana)* - constraints on the evolution of lizard life histories. J Exp Zool.

[7] Ketterson E, Nolan V, Cawthorn M, Parker P, Ziegenfus C (1996). Phenotypic engineering: using hormones to explore the mechanistic and functional bases of phenotypic variation in nature. Ibis.

[8] Zera AJ, Harshman LG (2001). The physiology of life history trade-offs in animals. Annu Rev Ecol Syst.

[9] Flatt T, Tu MP, Tatar M (2005). Hormonal pleiotropy and the juvenile hormone regulation of Drosophila development and life history. Bioessays.

[10] Williams T (2012). Hormones, life-history, and phenotypic variation: opportunities in evolutionary avian endocrinology. Gen Comp Endocrinol.

[11] Sapolsky RM, Romero LM, Munck AU (2000). How do glucocorticoids influence stress responses? Integrating permissive, suppressive, stimulatory, and preparative actions. Endocr Rev.

[12] Love OP, Breuner CW, Vezina F, Williams TD (2004). Mediation of a corticosterone-induced reproductive conflict. Horm Behav.

[13] Harshman LG, Zera AJ (2007). The cost of reproduction: the devil in the details. Trends Ecol Evol.

[14] Crespi EJ, Williams TD, Jessop TS, Delehanty B (2013). Life history and the ecology of stress: how do glucocorticoid hormones influence life-history variation in animals?. Funct Ecol.

[15] Wingfield JC, Romero LM, McEwen BS, Goodman HM (2001). Adrenocortical responses to stress and their modulation in free-living vertebrates. Handbook of physiology; section 7: the endocrine system coping with the environment: neural and endocrine mechanisms.

[16] Romero LM, Dickens MJ, Cyr NE (2009). The reactive scope model—A new model integrating homeostasis, allostasis, and stress. Horm Behav.

[17] Wingfield JC, Breuner C, Jacobs J, Etches RJ, Harvey S (1997). Corticosterone and behavioral responses to unpredictable events. Avian endocrinology.

[18] Landys MM, Ramenofsky M, Wingfield JC (2006). Actions of glucocorticoids at a seasonal baseline as compared to stress-related levels in the regulation of periodic life processes. Gen Comp Endocrinol.

[19] Silverin B (1986). Corticosterone-binding proteins and behavioral effects of high plasma levels of corticosterone during the breeding period in the pied flycatcher. Gen Comp Endocrinol.

[20] Bonier F, Moore I, Martin P, Robertson R (2009). The relationship between fitness and baseline glucocorticoids in a passerine bird. Gen Comp Endocrinol.

[21] Bonier F, Moore IT, Robertson RJ (2011). The stress of parenthood? Increased glucocorticoids in birds with experimentally enlarged broods. Biol Lett.

[22] Lancaster LT, Hazard LC, Clobert J, Sinervo BR (2008). Corticosterone manipulation reveals differences in hierarchical organization of multidimensional reproductive trade-offs in r-strategist and K-strategist females. J Evol Biol.

[23] Crossin G, Trathan P, Phillips R, Gorman K, Dawson A, Sakamoto K, Williams T (2012). Corticosterone predicts foraging behavior and parental care in macaroni penguins. Am Nat.

[24] Ouyang JQ, Muturi M, Quetting M, Hau M (2013). Small increases in corticosterone before the breeding season increase parental investment but not fitness in a wild passerine bird. Horm Behav.

[25] Bonier F, Martin PR, Moore IT, Wingfield JC (2009). Do baseline glucocorticoids predict fitness?. Trends Ecol Evol.

[26] Hau M, Ricklefs RE, Wikelski M, Lee KA, Brawn JD (2010). Corticosterone, testosterone and life-history strategies of birds. Proc R Soc B Biol Sci.

[27] Lendvai AZ, Bókony V, Angelier F, Chastel O, Sol D (2013). Do smart birds stress less? An interspecific relationship between brain size and corticosterone levels. Proc R Soc B Biol Sci.

[28] Winkler D, Luo M, Rakhimberdiev E (2013). Temperature effects on food supply and chick mortality in tree swallows (Tachycineta bicolor). Oecologia.

[29] Keil C, Röpnack A, Craig GC, Schumann U (2008). Sensitivity of quantitative precipitation forecast to height dependent changes in humidity. Geophys Res Lett.

[30] Thierry A-M, Massemin S, Handrich Y, Raclot T (2013). Elevated cort levels and severe weather conditions decrease parental investment of incubating Adélie penguins. Horm Behav.

[31] Spée M, Marchal L, Lazin D, Le Maho Y, Chastel O, Beaulieu M, Raclot T (2011). Exogenous corticosterone and nest abandonment: a study in a long-lived bird, the Adélie penguin. Horm Behav.

[32] Frich P, Alexander LV, Della-Marta P, Gleason B, Haylock M, Tank A, Peterson T (2002). Observed coherent changes in climatic extremes during the second half of the twentieth century. Clin Res.

[33] Barta Z, Houston Alasdair I, McNamara John M, Székely T (2002). Sexual conflict about parental care: the role of reserves. Am Nat.

[34] Schwagmeyer PL, Mock DW, Parker GA (2002). Biparental care in house sparrows: negotiation or sealed bid?. Behav Ecol.

[35] Dakin R, Lendvai AZ, Ouyang JQ, Moore IT, Bonier F (2015). Plumage colour is associated with partner parental care in mutually ornamented tree swallows.

[36] Hinde CA (2006). Negotiation over offspring care? -a positive response to partner-provisioning rate in great tits. Behav Ecol.

[37] Pogány Á, Szentirmai I, Komdeur J, Székely T (2008). Sexual conflict and consistency of offspring desertion in Eurasian penduline tit Remiz pendulinus. BMC Evol Biol.

[38] Ouyang JQ, Quetting M, Hau M (2012). Corticosterone and brood abandonment in a passerine bird. Anim Behav.

[39] Székely T (1996). Brood desertion in Kentish Plover Charadrius alexandrinus: an experimental test of parental quality and remating opportunities. Ibis.

[40] Webb JN, Székely T, Houston AI, McNamara JM (2002). A theoretical analysis of the energetic costs and consequences of parental care decisions. Philos Trans R Soc Lond Ser B Biol Sci.

[41] Butler RW (1988). Population dynamics and migration routes of Tree Swallows, *Tachycineta bicolor*, in North America. J Field Ornithol.

[42] Shutler D, Hussell DJT, Norris DR, Winkler DW, Robertson RJ, Bonier F, et al. Spatiotemporal patterns in nest box occupancy by tree swallows across North America. Avian Conservation Ecology. 2012;7(3).

[43] McCarty JP (2002). The number of visits to the nest by parents is an accurate measure of food delivered to nestlings in Tree Swallows. J Field Ornithology.

[44] Rose AP (2009). Temporal and individual variation in offspring provisioning by tree swallows: a new method of automated nest attendance monitoring. Plos One.

[45] Bonier F, Martin PR, Wingfield JC (2007). Maternal corticosteroids influence primary offspring sex ratio in a free-ranging passerine bird. Behav Ecol.

[46] Peig J, Green AJ (2009). New perspectives for estimating body condition from mass/length data: the scaled mass index as an alternative method. Oikos.

[47] Fox J (2002). Cox proportional‐hazards regression for survival data.

[48] Nur N, Holmes AL, Geupel GR (2004). Use of survival time analysis to analyze nesting success in birds: an example using loggerhead shrikes. Condor.

[49] Beyersmann J, Schumacher M, Allignol A (2012). Time-dependent covariates and multistate models.

[50] Burnham KP, Anderson DR (2002). Model selection and multi-model inference: a practical information-theoretical approach.

